# Web-Based Knowledge Translation Tool About Pediatric Acute Gastroenteritis for Parents: Pilot Randomized Controlled Trial

**DOI:** 10.2196/45276

**Published:** 2023-05-25

**Authors:** Lisa Hartling, Sarah A Elliott, Matthew Munan, Shannon D Scott

**Affiliations:** 1 Department of Pediatrics University of Alberta Edmonton, AB Canada

**Keywords:** knowledge translation, pediatric acute gastroenteritis, emergency department, health care decision-making, multiple methods, randomized controlled trial, qualitative interviews, web-based tool

## Abstract

**Background:**

Acute gastroenteritis (AGE) in children is a leading cause of emergency department (ED) visits, resulting in substantial health care costs and stress for families and caregivers. The majority of pediatric AGE cases are caused by viral infections and can be managed at home using strategies to prevent dehydration. To increase knowledge of, and support health decision-making for, pediatric AGE, we developed a knowledge translation (KT) tool (fully automated web-based whiteboard animation video).

**Objective:**

The aim of this study was to assess the potential effectiveness of the web-based KT tool in terms of knowledge, health care decision-making, use of resources, and perceived benefit and value.

**Methods:**

A convenience sample of parents was recruited between December 18, 2020, and August 10, 2021. Parents were recruited in the ED of a pediatric tertiary care hospital and followed for up to 14 days after the ED visit. The eligibility criteria included parent or legal guardian of a child aged <16 years presenting to the ED with an acute episode of diarrhea or vomiting, ability to communicate in English, and agreeable to follow-up via email. Parents were randomized to receive the web-based KT tool (intervention) about AGE or a sham video (control) during their ED visit. The primary outcome was knowledge assessed before the intervention (baseline), immediately after the intervention, and at follow-up 4 to 14 days after ED discharge. Other outcomes included decision regret, health care use, and KT tool usability and satisfaction. The intervention group participants were invited to participate in a semistructured interview to gather additional feedback about the KT tool.

**Results:**

A total of 103 parents (intervention: n=51, 49.5%, and control: n=52, 50.5%) completed the baseline and postintervention assessments. Of these 103 parents, 78 (75.7%; intervention: n=36, 46%, and control: n=42, 54%) completed the follow-up questionnaire. Knowledge scores after the intervention (mean 8.5, SD 2.6 vs mean 6.3, SD 1.7; *P*<.001) and at follow-up (mean 9.1, SD 2.7 vs mean 6.8, SD 1.6; *P*<.001) were significantly higher in the intervention group. After the intervention, parents in the intervention group reported greater confidence in knowledge than those in the control group. No significant difference in decision regret was found at any time point. Parents rated the KT tool higher than the sham video across 5 items assessing usability and satisfaction.

**Conclusions:**

The web-based KT tool improved parental knowledge about AGE and confidence in their knowledge, which are important precursors to behavior change. Further research is needed into understanding what information and delivery format as well as other factors influence parents’ decision-making regarding their child’s health.

**Trial Registration:**

ClinicalTrials.gov NCT03234777; https://clinicaltrials.gov/ct2/show/NCT03234777

**International Registered Report Identifier (IRRID):**

RR2-10.1186/s40814-018-0318-0

## Introduction

### Background

Acute gastroenteritis (AGE), characterized by frequent diarrhea or vomiting, is a leading cause of morbidity in children aged <5 years [1]. The majority of pediatric AGE cases are caused by viral infections and can be managed at home using oral rehydration strategies [2,3]. Despite this, AGE accounts for a large portion of children’s visits to the pediatric emergency department (ED), resulting in substantial health care costs, along with emotional and financial stress for the children’s parents and caregivers.

Parental knowledge about management of pediatric AGE has been shown to be low [4] and is often related to accessibility of health care information [5]. Parents have expressed a desire for information on AGE management strategies, such as identifying symptoms and treatment as well as understanding the normal course of illness and causes of AGE [6]. To address this gap, we codeveloped with parents a web-based knowledge translation (KT) tool in the form of a whiteboard animation video [7]. The video presents evidence-based home management strategies and identification of dehydration symptoms in the form of a narrative story that builds on parents’ reported experiences of having a child with AGE [8]. Storytelling has been shown to be effective for communicating health care information and influencing behavior [9-12]. In addition, video instructions may be more effective than written instructions at increasing knowledge of caregivers of children in the ED [13] and influencing behavior [14]. Finally, around the world, the prevalence of web-based health information searching by parents is high, with recent studies reporting a prevalence of 75% to 90% [15].

To date, there is little research on the effectiveness of web-based KT tools that deliver evidence-based health information to parents. Previous studies exploring the effect of KT tools on parent knowledge, experience, and health behavior have reported mixed results [16]. However, a recent study has demonstrated the effectiveness of a KT tool that was relevant to parents’ needs and built confidence to increase parent-reported uptake of evidence-based strategies [17]. It is hypothesized that providing information to parents empowers them to make effective health care decisions (eg, when to seek medical care or go to the ED) and reduces health system costs [18].

### Objectives

Undertaking a rigorous effectiveness evaluation is often resource intensive. Nevertheless, it is essential to understand whether KT tools are effective for their intended audience before making investments in large-scale implementation. The primary aim of this pilot randomized controlled trial (NCT03234777) was to assess the potential effectiveness of a web-based KT tool for pediatric AGE in terms of parent knowledge, confidence in knowledge, decision regret, health care use, and usability and satisfaction. We embedded a qualitative study to gather a greater understanding of parents’ perceptions of the KT tool. The results from this usability trial involving the intended end users will be important to inform future tools and optimize investments in research and KT.

## Methods

### Ethics Approval

The study was approved by the University of Alberta health research ethics board (Pro00091675) before commencement.

### Scientific Outcomes

The protocol for this parallel-group 2-arm pilot randomized controlled trial was published [19], and the study was registered at ClinicalTrials.gov (NCT03234777). The trial collected scientific (potential effectiveness) and feasibility outcomes [19]; this paper reports on the scientific outcomes. No changes to the web-based intervention or methods were made after trial commencement. Refer to Multimedia Appendix 1 for the CONSORT (Consolidated Standards of Reporting Trials) eHEALTH checklist.

### Setting and Population

Recruitment occurred between December 18, 2020, and August 10, 2021. A convenience sample of parents or legal guardians of children aged <16 years presenting to the ED with diarrhea or vomiting were invited. Parents had to be fluent in English and agree to be contacted via email for follow-up. Parents were not eligible if the children had chronic gastrointestinal problems or inflammatory bowel disease (ie, Crohn disease or ulcerative colitis), were taking immunosuppressive therapy or had known immunosuppression, had undergone oral or gastrointestinal surgery in the preceding 7 days, or had had a prior ED visit for vomiting or diarrhea in the preceding 14 days.

### Intervention and Control

The intervention (KT tool) was a web-based 3-minute whiteboard animation video codeveloped by the research team and parents and caregivers and tested with stakeholders (Multimedia Appendix 2) [8]. The video content was informed by research evidence for the treatment and management of pediatric AGE [20]. The control intervention (sham video) was a 3-minute video about hand hygiene produced by the US Centers for Disease Control and Prevention, a reputable government health agency [21]. This resource was chosen with input from a pediatric infectious disease physician and a pediatric emergency physician. It was felt that an active control intervention was preferable so that all participants received some evidence-based information. The content of the control video has widespread relevance and is important for infection control but did not overlap in content with the intervention video.

### Process

**Figure 1:**
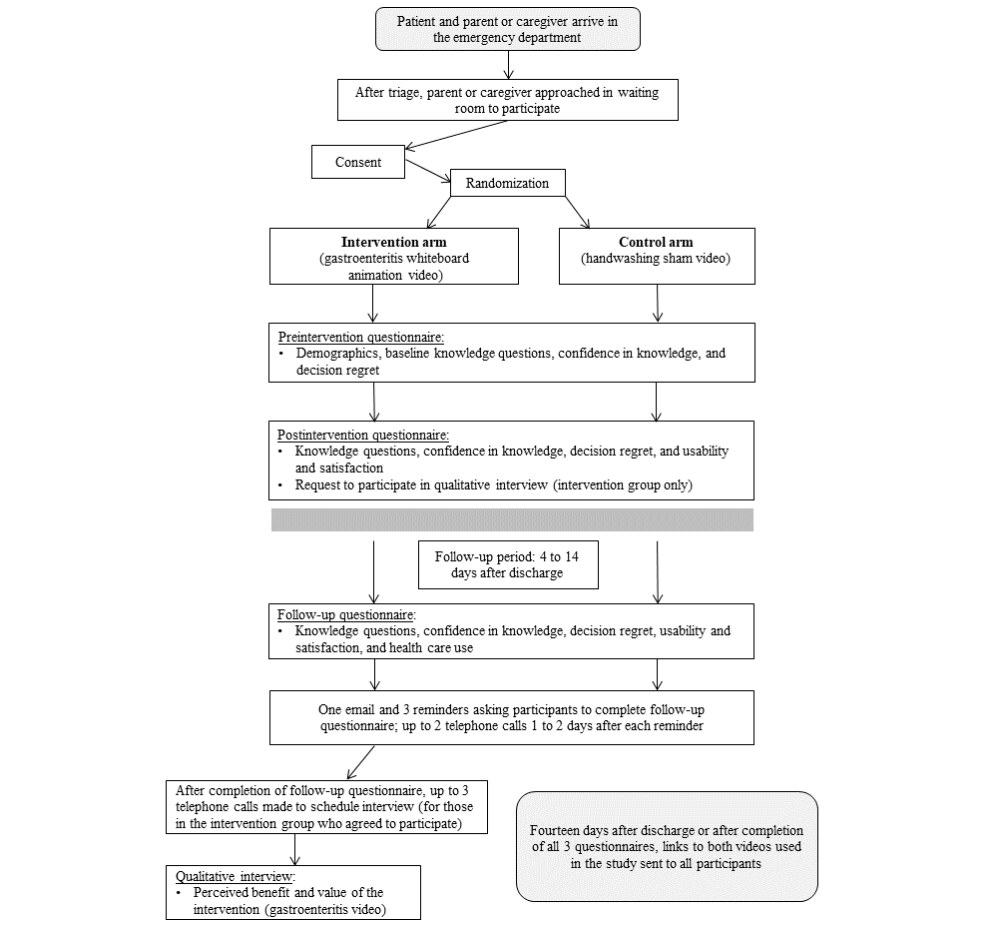
Trial flow and timing of data collection.

Figure 1 provides details regarding study process and participant flow. Parents or caregivers of children presenting with vomiting or diarrhea were approached by a research coordinator in the ED waiting room after triage. Research coordinators explained the study process and, if they were interested, parents were handed an iPad containing a web-based application developed by Nooro Inc. The web-based application was used to capture implied consent (by overt action), randomly assign participants to groups, deliver the intervention, and collect data in the ED. This occurred while the parent was in the waiting room before their child received treatment. The same web-based application was used to capture follow-up questionnaire data and automatically send reminders to participants. The web-based application was pilot tested to identify and fix any issues before recruitment.

Parents were randomly assigned (1:1 ratio) to view the KT tool or sham video. A randomization table was generated by a statistician using a computer program with an alternating block balanced approach with block sizes of 4. Research personnel and participants were unaware of the next group assignment. Parents and research personnel were blinded to the interventions being compared.

Parents completed a web-based preintervention baseline questionnaire assessing knowledge about AGE, confidence in knowledge, and decision regret. Immediately after viewing the intervention and control, participants completed a postintervention web-based questionnaire. This questionnaire repeated knowledge, confidence in knowledge, and decision regret questions, and included questions about usability and satisfaction regarding the intervention. Participants were automatically sent a follow-up web-based questionnaire by email 4 days after the ED visit (the date on which the baseline questionnaire was completed). This questionnaire repeated knowledge and decision regret questions and contained additional questions on health care use after the ED visit and intervention usability. Research personnel contacted the parents by telephone every 3 days to remind them to complete the follow-up questionnaire. Parents had 10 days to complete this questionnaire.

### Outcomes

Outcomes, methods of assessment, and timing of assessment are listed in Table 1. The primary outcome was change in parental knowledge measured using an 8-item questionnaire informed by the abridged Caregiver Gastroenteritis Knowledge Questionnaire [22]. The questionnaire was tailored to the information in the intervention and pilot tested with parents. Scores are summed and range from 0 to 13, with higher scores indicating a higher level of knowledge (ie, more correct answers; Multimedia Appendix 3). The secondary outcomes included parental decision regret, confidence in knowledge, post-ED visit health care use, and perceived benefit and value of the intervention. Decision regret was assessed using a validated decision regret tool [23]. Scoring involved reversing the scores of the 2 negatively phrased items, then taking the mean of the 5 items, with higher numbers indicating greater regret (Multimedia Appendix 4 [23]). Parents’ self-reported confidence in knowledge was assessed using a 5-point Likert scale (ranging from 1=very confident to 5=very not confident). Post-ED visit health care use was captured by asking parents whether they had to bring their child back to the ED for vomiting or diarrhea or seek additional care from another health professional since the initial ED visit. Usability and satisfaction of the interventions were assessed based on responses to 5 statements using a 5-point Likert scale (ranging from 1=strongly agree to 5=strongly disagree). The perceived benefit and value of the web-based intervention were further explored through qualitative interviews with a subset of parents who received the KT tool.

**Table 1:** Study outcomes, method of assessment, and timing of assessment.

Outcome		Method of assessment	Timing of assessment^a^
Primary: parental knowledge of gastroenteritis		Eight questions about gastroenteritis, symptoms, and management based on the abridged Caregiver Gastroenteritis Knowledge Questionnaire	Repeated 3 times: Baseline After the intervention Follow-up
**Secondary**			
	Parental decision regret	Validated scale used to assess “remorse or distress over a decision”	Repeated 3 times: Baseline After the intervention Follow-up
	Parental confidence in knowledge rating	A 5-point Likert scale ranging from 1=very confident to 5=very not confident	Repeated 3 times: Baseline After the intervention Follow-up
	Post-ED^b^ visit health care use	Two yes or no questions asking whether parents went back to the ED or contacted health care professional and qualitative interview questions	Asked once at follow-up and during qualitative interview (up to 3 months after the ED visit)
	Usability and satisfaction	Five questions related to the perceived benefit of the KT^c^ tool and influence on knowledge and decision-making	Repeated 2 times: After the intervention Follow-up

^a^Baseline: in the ED before receiving the intervention, after the intervention: in the ED after receiving the intervention, and follow-up: 4 to 10 days after the ED visit.

^b^ED: emergency department.

^c^KT: knowledge translation.

### Qualitative Interviews

The perceived benefit and value of the KT intervention were also evaluated through qualitative interviews, which were conducted to help understand and contextualize the results of the trial and provide more detailed feedback from participants about their experience with, and perceptions of, the intervention [24,25]. The interviews allowed parents to elaborate on elements of the KT tools that were not assessed via questionnaires and to provide in-depth details about their experience of managing a child with AGE. At the end of the last follow-up questionnaire, parents were asked about their willingness to participate in a 30- to 60-minute one-on-one web-based interview. The semistructured interview guide was developed by the research team and pilot tested with parents (available on request). The interview questions moved from the general to the specific regarding the KT tool, with interviews later in the data collection period becoming increasingly more focused [26] on certain aspects of the KT tool (eg, usefulness of messaging, timing of receiving the KT tool, and modality [ie, whiteboard animation video]). Parents were interviewed using Zoom videoconferencing software (Zoom Video Communications, Inc). The interviews were recorded and transcribed verbatim.

### Data Analysis

#### Sample Size

Because of the exploratory and pilot design of this study (ie, assessing potential effectiveness), no formal sample size calculation was required [27,28]. However, guided by previous research [27,28], we planned to recruit 100 parents (50 per group) to provide reasonable effect estimates to inform sample size calculations for future large-scale trials.

#### Quantitative Analysis

Statistical analysis was conducted in SPSS software (version 26.0; IBM Corp), with *P*<.05 considered statistically significant. Baseline demographic variables were described for each group using mean (SD) or median (IQR). Differences between the groups at baseline, after the intervention, and at follow-up were compared using independent 2-tailed *t* tests. Analysis was based on intention to treat and by original assigned groups.

#### Qualitative Analysis

The thematic analysis framework developed by Braun and Clarke [29] was used to review the transcripts, develop initial codes, identify themes, review themes, define and name themes, and report findings. Using NVivo (QSR International), line-by-line coding of interview data was conducted by the research team, and codes were developed. From these codes, a series of themes, relevant to the research questions, were generated. Interviews were performed until we had confidence that saturation had been reached and that variations of data had been sufficiently explored [30].

## Results

### Sample

**Figure 2:**
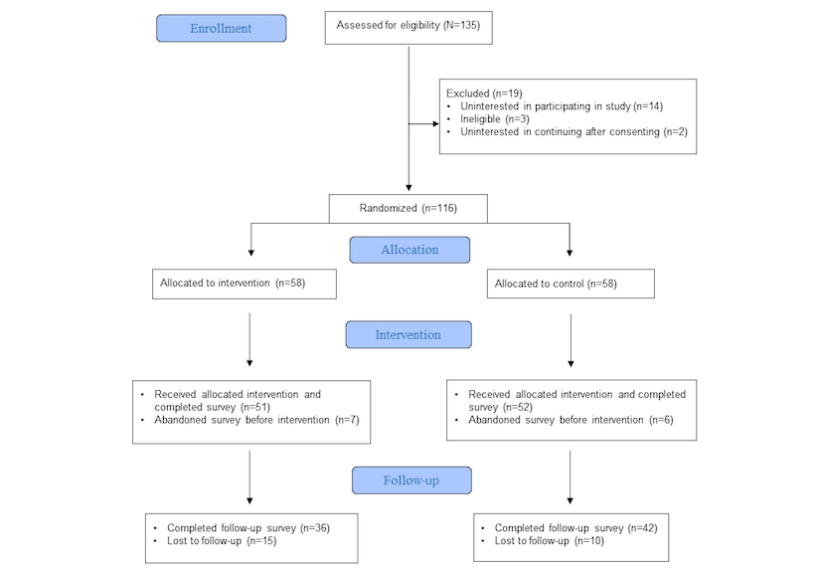
CONSORT (Consolidated Standards of Reporting Trials) flow diagram.

Of the 116 parents enrolled and randomized, 103 (88.8%) viewed the intervention and control and completed questionnaires in the ED (Figure 2). The groups were comparable at baseline (Table 2). Of the 36 parents who viewed the KT tool and completed all questionnaires, 12 (33%) participated in an interview.

**Table 2:** Description of study participants (N=103).

Variable		Intervention group (n=51), n (%)	Control group (n=52), n (%)
**Gender**			
	Woman	45 (88)	45 (87)
	Man	6 (12)	6 (12)
	Nonbinary	0 (0)	1 (2)
**Age (years)**			
	20-30	14 (27)	10 (19)
	31-40	29 (57)	31 (60)
	41-50	7 (14)	8 (15)
	≥51	1 (2)	3 (6)
**Ethnicity**			
	Arab	1 (2)	3 (6)
	Black or African	7 (14)	4 (8)
	Chinese	1 (2)	1 (2)
	Filipino	3 (6)	2 (4)
	Indigenous	2 (4)	1 (2)
	Latin American	2 (4)	4 (8)
	South Asian or Southeast Asian	3 (6)	8 (16)
	White	30 (59)	26 (50)
	Other	1 (2)	2 (4)
	Prefer not to answer	1 (2)	1 (2)
**Citizenship status**			
	Canadian by birth	32 (63)	32 (62)
	Canadian by naturalization	6 (12)	7 (13)
	Other country	10 (20)	12 (23)
	Prefer not to answer	3 (6)	1 (2)
**Relationship status**			
	Single	11 (22)	7 (14)
	Partnered	38 (75)	45 (87)
	Other	0 (0)	0 (0)
	Prefer not to answer	2 (4)	0 (0)
**Highest level of education**			
	Some high school or high school diploma	12 (24)	11 (21)
	Certificate or college	15 (30)	15 (29)
	Bachelor’s degree	11 (22)	12 (23)
	Graduate degree	5 (10)	8 (15)
	Other	1 (2)	1 (2)
	Prefer not to answer	7 (14)	5 (10)
**Parental role**			
	Mother	45 (88)	45 (87)
	Father	6 (12)	6 (11)
	Other	0 (0)	1 (2)
**Number of children**			
	1	16 (32)	20 (38)
	2	16 (32)	20 (38)
	3	11 (22)	11 (20)
	4-5	10 (16)	1 (2)
**Age (years) of child with AGE^a^**			
	<1	11 (22)	12 (23)
	1-4	23 (45)	21 (40)
	5-10	8 (16)	10 (19)
	>11	9 (18)	9 (17)
**Gender of child with AGE**			
	Girl	24 (47)	32 (62)
	Boy	27 (53)	20 (38)
**Did the child with AGE experience vomiting or diarrhea in the past?**			
	Yes	37 (71)	42 (82)
	No	15 (29)	9 (18)
**Current symptom start time**			
	Today	16 (32)	16 (31)
	1-2 days ago	11 (22)	14 (27)
	3-5 days ago	12 (24)	11 (21)
	≥6 days ago	8 (16)	10 (19)
	Other	4 (8)	1 (2)
**Number of vomits in last 24 hours**			
	None	7 (14)	10 (19)
	1-5	29 (57)	22 (42)
	6-10	9 (18)	11 (21)
	>10	6 (12)	9 (17)
**Number of episodes of diarrhea in last 24 hours**			
	None	27 (53)	32 (62)
	1-5	22 (43)	13 (25)
	6-9	1 (2)	4 (8)
	≥10	1 (2)	3 (6)
**Other members of household with vomiting or diarrhea in the last month**			
	Yes	4 (8)	8 (15)
	No	46 (90)	43 (83)
	Unsure	1 (2)	1 (2)
**Did you contact a health professional before coming to the ED^b^ today?**			
	Yes	26 (51)	23 (44)
	No	25 (49)	29 (56)
**Did you look up information before coming to the** **ED** **today?**			
	Yes	30 (59)	25 (48)
	No	21 (41)	27 (52)

^a^AGE: acute gastroenteritis.

^b^ED: emergency department.

### Quantitative Outcomes

#### Knowledge

Baseline knowledge scores were similar between the groups (Table 3). Postintervention knowledge scores were significantly higher in the intervention group than in the control group (mean 8.5, SD 2.6 vs mean 6.3, SD 1.7, respectively; *P*<.001). On follow-up (4-14 days after the ED visit), this difference remained, with the intervention group retaining higher knowledge scores (mean 9.1, SD 2.7 vs mean 6.8, SD 1.6; *P*<.001). No change in parents’ self-reported confidence in knowledge was observed in the control group at any time point. In the intervention group, parents’ confidence in knowledge increased from baseline to after the intervention (mean change 0.7, 95% CI 0.5-0.9; *P*<.001) but decreased from after the intervention to follow-up (mean change −0.3, 95% CI −0.5 to −0.1; *P*<.001). Overall, after the intervention, the intervention group reported greater confidence in knowledge than the control group. At follow-up, there was no difference between the groups (Table 3).

**Table 3:** Comparison of primary and secondary outcomes.

Outcome and timing			Intervention group (n=51^a^), mean (SD)	Control group (n=52^a^), mean (SD)	Mean difference (SE)	*P* value
**Knowledge score (score range 0-13; higher scores=more correct answers)**						
		Baseline	6.4 (2.0)	6.6 (1.9)	0.2 (0.4)	.53
		After the intervention	8.5 (2.6)	6.3 (1.7)	2.3 (0.4)	<.001
		Follow-up	9.1 (2.7)	6.8 (1.6)	2.3 (0.5)	<.001
**Confidence in knowledge^b^ (ranging from 1=very confident to 5=very not confident)**						
		Baseline	2.6 (0.7)	2.2 (0.7)	0.4 (0.1)	.01
		After the intervention	1.9 (0.6)	2.2 (0.7)	0.3 (0.1)	.05
		Follow-up	2.2 (0.7)	2.2 (0.6)	0.02 (0.1)	.87
**Decision regret (score range 1-5; higher numbers=greater regret)**						
		Baseline	1.8 (0.6)	1.7 (0.7)	0.1 (0.1)	.64
		After the intervention	1.8 (0.8)	1.7 (0.7)	0.0 (0.1)	.75
		Follow-up	1.7 (0.7)	1.8 (0.7)	0.1 (0.2)	.64
**Usability and satisfaction (ranging from 1=strongly agree to 5=strongly disagree)**						
	**Video increased knowledge about AGE^c^**					
		After the intervention	1.8 (0.9)	3.1 (1.4)	1.2 (0.2)	<.001
		Follow-up	1.9 (0.7)	3.1 (1.2)	1.2 (0.2)	<.001
	**Would use video to make decisions about child’s health**					
		After the intervention	1.8 (0.9)	2.7 (1.3)	1.1 (0.2)	<.001
		Follow-up	1.8 (0.8)	3.0 (1.2)	1.2 (0.2)	<.001
	**Video provided useful information about AGE**					
		Follow-up	1.7 (0.6)	2.9 (1.2)	1.2 (0.2)	<.001
	**Video increased confidence in managing AGE**					
		Follow-up	1.9 (0.8)	3.0 (1.1)	1.1 (0.2)	<.001
	**Would recommend video to others**					
		Follow-up	1.8 (0.7)	2.8 (1.3)	1.0 (0.2)	<.001

^a^Numbers reflect participants who completed baseline and postintervention questionnaires; 71% (36/51) and 81% (42/52) of the participants completed follow-up questionnaires among the intervention and control groups, respectively.

^b^Knowledge and decision regret change were self-reported by participants.

^c^AGE: acute gastroenteritis.

#### Decision Regret

Baseline decision regret scores indicated a low level of decision regret for both groups (Table 3), with no change in decision regret scores in either group after the intervention or at follow-up.

#### Post-ED Visit Health Care Use

Of those who completed the follow-up questionnaire, 5 (14%) of the 36 parents in the intervention group and 3 (7%) of the 42 parents in the control group returned to the ED within 4 to 14 days after ED discharge. Overall, 11 (31%) of the 36 parents in the intervention group and 9 (21%) of the 42 parents in the control group sought health care outside the ED: 64% (7/11) of the parents in the intervention group and 78% (7/9) of the parents in the control group made a visit to their pediatrician or family physician, 22% (2/9) of the parents in the control group attended a specialized clinic or communicated with an allied health professional, 9% (1/11) of the parents in the intervention group called the regional health services telehealth support line, and 27% (3/11) of the parents in the intervention group admitted their child to hospital.

#### Perceived Benefit and Value of the KT Tool

There were significant differences in perceived benefit and value of the KT tool compared with the control. Parents who received the KT tool felt more strongly that the tool increased their knowledge, and they were more confident in being able to manage and care for their child if they had AGE again. Compared with the control, parents found the KT tool useful, would use it to make health care decisions for the child, and recommend it to others.

### Qualitative Interviews

#### Overview

Three major themes emerged regarding parents’ experiences of seeking care for AGE and usefulness of the KT tool (Table 4).

**Table 4:** Thematic analysis of the semistructured interviews.

Theme and subtheme		Representative quote
**Parents seek information from a variety of sources before going to the ED^a^**		
	Web-based sources	“When we try to make a decision based on their health, of course we access the Health Link^b^ and then the online resources” (participant #6).
	Contacting health professionals	“My doctor just wasn’t available. And we couldn’t get any advice from the nursing staff. We had to see the doctor. So, I ended up calling Health Link just to see what I could do to help her from home and they requested that we go in” (participant #9).
	Contacting friends and family	“Often times we can call our moms or our grandmas and ask them” (participant #5).
**KT^c^ tool is relevant and increased knowledge**		
	KT tool is useful if viewed before going to the ED	“I knew about gastroenteritis, even I did know there was viral gastroenteritis, I thought it was bacteria so I learned about viral...then of course the key drugs if it’s manageable, Tylenol and Pedialyte. Pedialyte is not really a drug but so I learnt about that. Then at the end I also learnt that if it doesn’t seem like it’s getting worse then you can wait 2 or 3 days, then I would have called his doctor instead” (participant #2).
	Parents would use the KT tool and share it with others	“I would definitely share it with others because I know of a few people who would automatically just assume oh my child’s dehydrated and I’ll just take him straight to the emergency room. Now actually watching it, it explains very well what you should be looking for and when your child needs extra medical attention” (participant #4).
	KT tool is not applicable to all children	“The signs of dehydration and then when to go to emergency. Again, I think that’s applicable for children maybe 6 months and older” (participant #1).
**KT** **tool influence on decision-making**		
	KT tool would not change decision to go to the ED	“No, because I was already recommended to go there, it wasn’t a choice that I made solely on my own. I did it with the recommendation” (participant #1).
	Access to the KT tool made participants question their decision	“My biggest concern originally is that she was so little and so, so young. And that’s why I didn’t want to take any chances. But I guess that video just helped clear up. But not to be worried. Unless I see these symptoms, then I can react. And then, you know, calling 811^b^ was the right thing to do. And probably just scheduling a doctor’s visit would have been just, you know, probably the proper thing instead of running to emergency right away” (participant #7).
	KT tool would help make decisions in the future	“I would actually go through the video and look for those kinds of symptoms and see what I could do at home first before automatically assuming he has to go back to the emergency room” (participant #4).

^a^ED: emergency department.

^b^Health Link and 811 refer to a free 24/7 telephone service that provides nurse advice and general health information for Albertans.

^c^KT: knowledge translation.

#### Parents Seek Information From Various Sources Before Going to the ED

Parents sought information from a variety of sources before making the decision to take their child to the ED. Parents looked for web-based information through official websites such as the regional health service provider or WebMD. The majority of the parents indicated that they contacted health professionals for medical advice to inform their decisions, which included using the regional telehealth line (a 24/7 nonemergent health line staffed by registered nurses), calling their pediatrician, or going to a walk-in clinic. Several of the parents sought advice or information from family members (eg, mothers and grandmothers) before going to the ED.

#### The KT Tool Is Relevant and Increases Knowledge

Overall, parents described learning important information from the KT tool and indicated that the video was relevant to their situation. They thought that the KT tool would be most useful if viewed before going to the ED. Parents reported that they would use the video and share it with others and that they found the information clear and concise. Some of the parents felt that the video was not applicable to all children (eg, the dehydration checklist as well as signs and symptoms were not relevant to infants); therefore, multiple tools for children of different ages may be appropriate.

#### KT Tool Influence on Decision-making

Parents expressed that the KT tool may influence decision-making if viewed before going to the ED but that the video would only supplement decision-making and, in most cases, would not be the deciding factor. Parents indicated that although the video was useful and provided good information, it would not have influenced their decision to go to the ED. In most cases, they had already called a helpline or received other advice to go to the ED. However, parents felt that the information provided in the video reinforced their decision to take their child to the ED. A subset of parents indicated that the video made them question whether going to the ED was the right decision or whether they could have managed their child’s symptoms at home.

Although the video did not influence most decisions, parents expressed that having access to the video before going to the ED would influence future decisions on treatment and management of AGE. Moreover, a web-based tool would increase accessibility. Parents shared that they would watch the video and use the information to inform treatment decisions, including when to go to the ED.

## Discussion

### Principal Findings

Connecting parents and families to effective evidence-based resources has the potential to improve knowledge and confidence in decision-making, which may subsequently reduce health care use and costs. We found that the KT tool codeveloped with parents and tailored to their needs increased their knowledge; however, the intervention had no effect on parents’ regret regarding their decision to take their child to the ED. These findings suggest that increasing knowledge may not be sufficient to change behavior (decisions and actions by parents to seek emergency care for their child). It is well established in behavioral research that knowledge is only 1 factor that drives behavior, for example, the capability, opportunity, motivation, and behavior (COM-B) model outlines different elements that influence behavior, including physical capability, psychological capability, reflective motivation, automatic motivation, and physical and social opportunity [31]. Knowledge and understanding can affect psychological capability and reflective motivation, but other inputs are required to affect the other components that influence behavior. Parents are not rational actors; our findings highlighted that parents’ decisions are shaped by a multitude of potentially interacting factors, including seeking information from a variety of sources. Although the KT tool scored favorably in terms of usability and satisfaction, parents indicated that the tool would only supplement decision-making and not be the determining factor. External elements (eg, calling a health helpline or asking a family member) were also drivers of behavior and decision-making.

Knowledge and experience of a childhood illness can also affect motivation; for instance, it has been shown that previous experience can influence a parent’s ability to determine the severity of their child’s illness and make a decision regarding their care [32]. McWilliams et al [33] found that a standardized education program for parents on acute otitis media was associated with a reduction in ED visits for otitis media in the year after the intervention. By increasing parental knowledge on treatment and management strategies for childhood illnesses such as AGE, parents may be better equipped to make future decisions regarding their child’s health and when it is appropriate to take them to the ED.

Digital and web-based KT tools offer a promising approach to communicate complex health information to parents and support their health care decision-making; however, there are many aspects of this complex process that remain unclear; for instance, how and when to provide these tools to parents during the overwhelming situation of having a child who is acutely ill. It is also unclear how to sustain the effects of these tools to shape future health care interactions. A previous study by our group that assessed the effects of storytelling as a communication tool found that a KT tool in the form of story booklets had a small but statistically significant impact on decision regret [9]. Although the difference was statistically significant, it was unclear whether the change would be clinically important. Similarly, a recent study performed by Jove-Blanco et al [34] found that video discharge instructions improved parental knowledge but did not have an effect on ED revisits. Chande et al [35] also found that the effects of an education intervention were not sustained; as a result, ED use habits in the 6 months after the ED visit were not altered.

Around the globe, many jurisdictions are developing approaches to reduce avoidable ED visits to improve appropriateness of care. However, many educational interventions seem to have had little to no effect on initial or subsequent ED visits [34,36,37]. The decision to take a child to the ED is complex, often overwhelming, and may be informed by a variety of different sources such as internet searches, consultation with family members, or communication with health care professionals [38]. In this study, half of the parents reported contacting a health professional (49/103, 47.6%) or looking up information on the web before going to the ED (55/103, 53.4%).

It is difficult to determine what a potentially avoidable ED visit is compared with a necessary visit for pediatric AGE. Measuring appropriateness of pediatric ED visits is challenging [39]. A recent study found low levels of agreement among clinicians on what constitutes an appropriate visit [39]. In our interviews, some of the parents stated that they were instructed by health care providers to take their child to the ED, whereas others referenced “following their gut” to make the decision. Although the information presented in the KT tool may be relevant and informative regarding the parent’s current situation, it may not carry the same influence as a conversation with a health care provider or “gut feelings.” The interviews provided additional illumination, with parents indicating that viewing the KT tool would not influence their decision if a health care provider had already advised taking their child to the ED; however, if viewed before the decision was made, it could be useful (likely in combination with other sources) to decide whether an ED visit was necessary.

Overall, parents provided positive feedback on the KT tool, indicating that it is useful, increased knowledge, and could be used to help with decision-making about their child’s health. This is consistent with our previous usability testing in which the tool was described as “informative, clear, and to the point” and that the checklists in the video are helpful for decision-making [8]. When asked about the best context in which to view the KT tool, parents indicated that the tool would be most helpful if viewed before going to the ED, and suggestions for dissemination included prenatal classes or regular clinic visits. Furthermore, having a web-based KT tool supports access when parents need information.

### Future Research

Health research funding agencies are prioritizing and making large investments in KT (or knowledge mobilization). Nevertheless, it is essential to conduct rigorous evaluations to understand whether KT efforts and interventions are having their intended impact, as well as how and when they need to be delivered. This trial with a qualitative component provides an important starting point for understanding the potential effectiveness of a KT tool codeveloped with parents. We found an impact on knowledge and parents’ confidence in their knowledge, which are important precursors to decision-making and behavior change [40,41]. A limitation of this trial is that parents had already made the decision to take their child to the ED. An important next step will be to share KT tools with parents before they make the decision to seek care to assess the impact of the KT tools on decision-making and service use. Mixed methods approaches using both quantitative and qualitative data collection will be important to understand impacts as well as contextualize *how* the KT tools affect the decision-making process. Continued research to understand the impact of web-based KT tools and their features that influence decision-making and use of health care services is critical. Furthermore, evaluation with a more diverse population (in terms of demographics and web literacy) would inform generalizability.

### Strengths and Limitations

Few KT interventions for parents and patients undergo rigorous effectiveness evaluations, which is a key strength of our study. Although we evaluated effectiveness using a randomized and blinded design, this was a pilot trial and may not have had the power to detect differences for some of our secondary outcomes, in particular, decision regret. However, our data will allow for sample size calculations for future trials in this area. Another key limitation is the timing of delivering the intervention. Because of practical considerations, we recruited parents in the ED after they had already made the decision to bring their child. We assessed decision regret using a validated tool; however, this may not capture the impact a KT tool could have on decision-making before going to the ED.

It is important to note that the results might not be generalizable across parenting roles, given the higher percentage of mothers participating in the study; however, this is similar to previous child health studies, which report that mothers assume a care provider role and more often participate in child health research [42-44]. Because of the sample size, there may have been some imbalances between the groups with respect to some characteristics (eg, the intervention group had more participants with 4-5 children). It is unclear how this would have influenced the results; however, there were no differences at baseline between the groups with respect to the primary outcome of knowledge. In addition, loss to follow-up, particularly for the postintervention questionnaire (although similar across the groups; 10% difference), may have biased the results; for instance, those who participated in the follow-up questionnaires and interviews may have been more engaged and overestimated the effects.

### Conclusions

This pilot randomized controlled trial showed that a KT tool codeveloped with parents compared with a sham control significantly increased parents’ knowledge about AGE. No differences were observed for decision regret, suggesting that other factors may affect the multifaceted decision-making process of when to seek ED care. The qualitative results offered important insights and underscored the idea that multiple factors influence parents’ decision-making when their child is sick. Although the KT tool scored favorably in terms of usability and satisfaction, parents indicated that the tool would only supplement decision-making and not be the determining factor. Further research into understanding what information and factors influence parents’ decision-making is warranted.

## Appendix

Multimedia Appendix 1 CONSORT-eHEALTH checklist (V 1.6.1).

Multimedia Appendix 2 Screenshots of the web-based knowledge translation tool (intervention).

Multimedia Appendix 3 Knowledge question scoring framework.

Multimedia Appendix 4 Decision regret scale (adapted from The Ottawa Hospital et al [23]).

## Abbreviations

AGE: acute gastroenteritis
COM-B: capability, opportunity, motivation, and behavior
CONSORT: Consolidated Standards of Reporting Trials
ED: emergency department
KT: knowledge translation

## Data Availability

Data are available upon reasonable request to the corresponding author.
