# Supplementary material for: Web-Based Knowledge Translation Tool About Pediatric Acute Gastroenteritis for Parents: Pilot Randomized Controlled Trial
Source: JMIR Form Res. 2023 May 25;7:e45276. doi: 10.2196/45276 (PMC10251226; doi:10.2196/45276)
Supplement: Multimedia Appendix 2 [file formative_v7i1e45276_app2.pdf]

CHILDHOOD VOMITING AND DIARRHEA (GASTROENTERITIS)

A web-based knowledge translation tool for parents

Hartling L, Elliott SA, Munan M, Scott SD. Web-based knowledge translation tool for parents about pediatric acute gastroenteritis: a pilot randomized trial. JMIR Form Res (forthcoming). doi:10.2196/45276 http://dx.doi.org/10.2196/45276

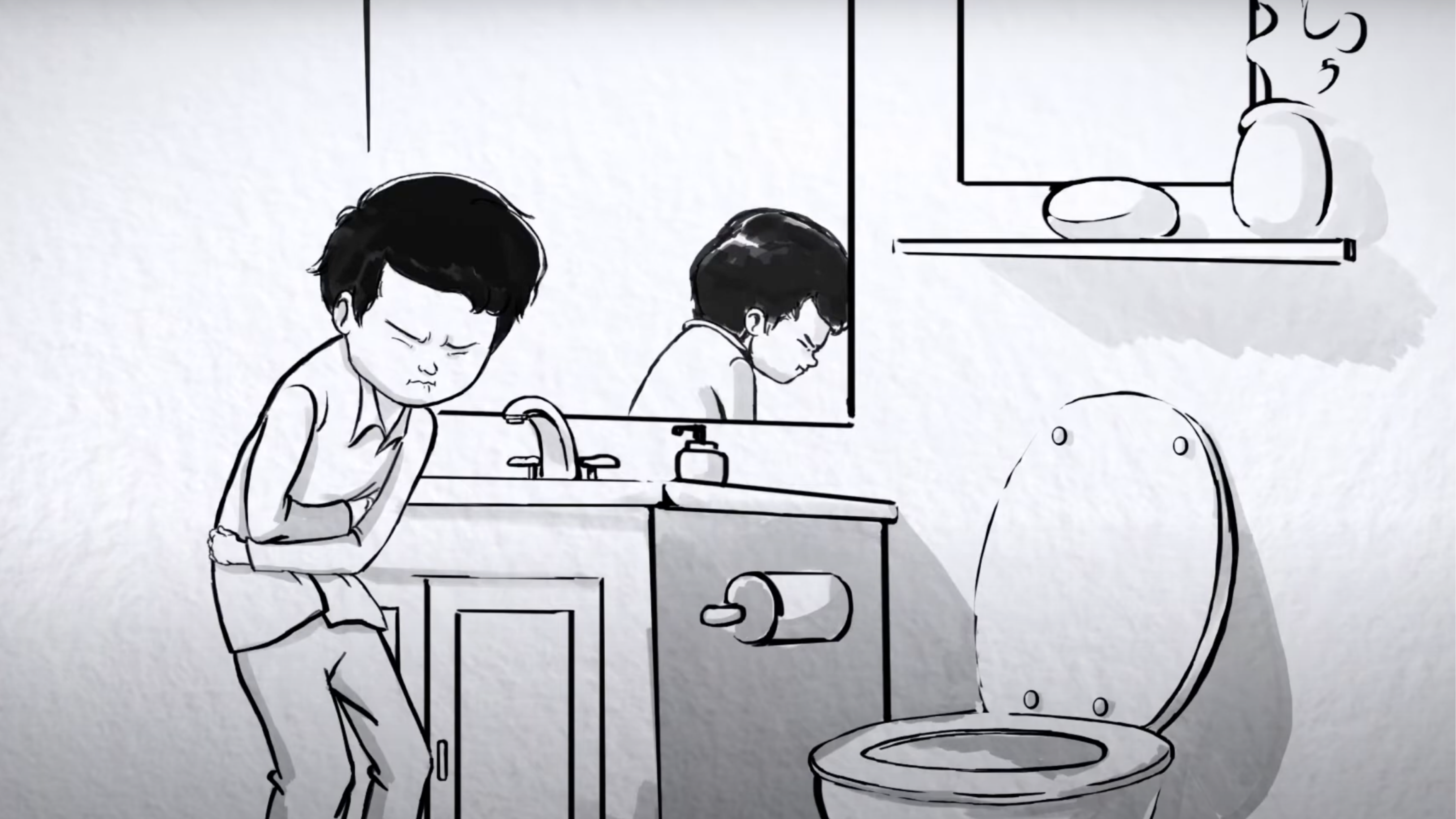

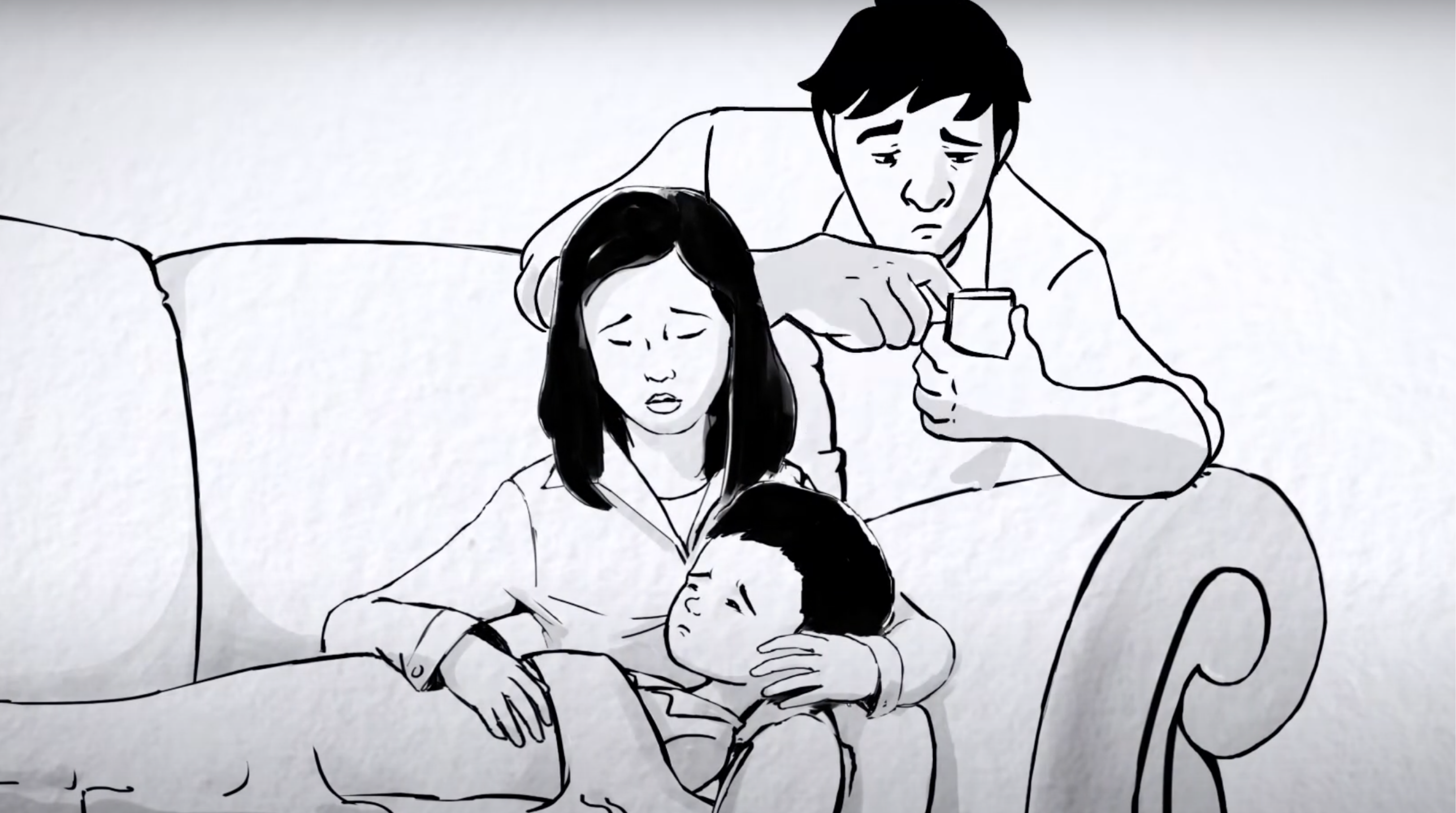

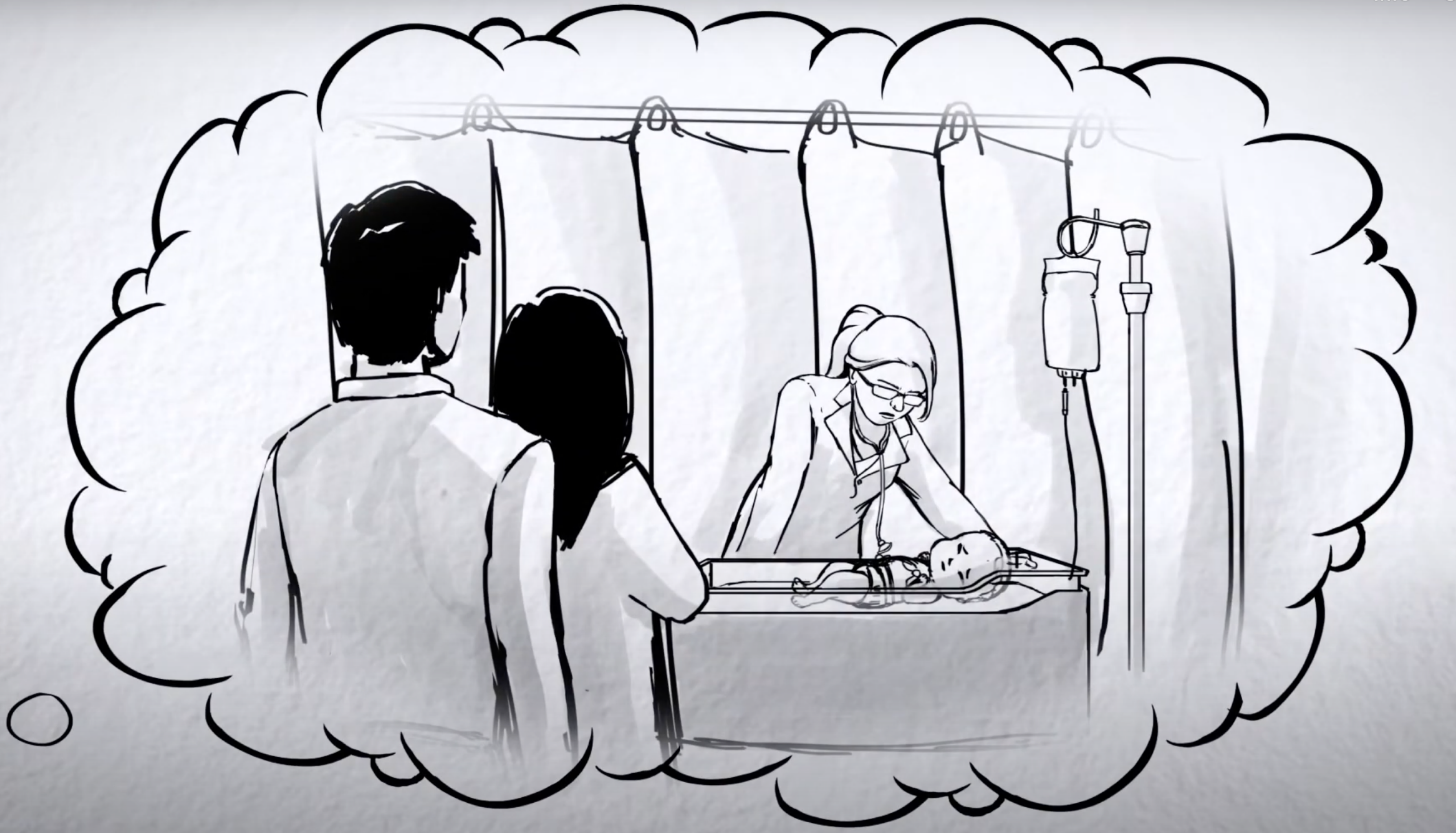

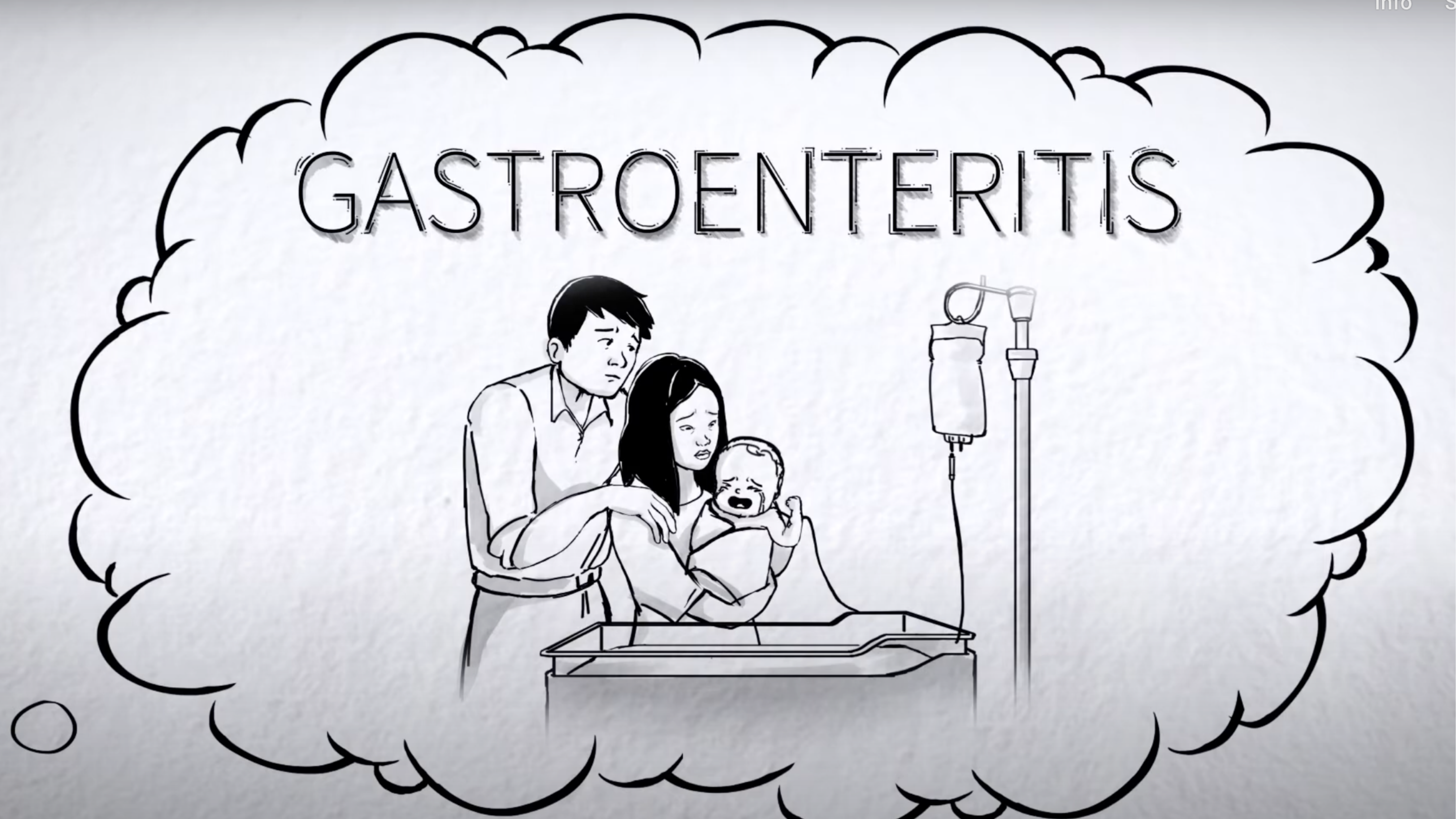
GASTROENTERITIS

VIRAL GASTROENTERITIS

How can I tell if my child is dehydrated?

Signs of dehydration include:

1. Thirst
2. No tears when crying
3. Sunken eyes
4. Dry mouth

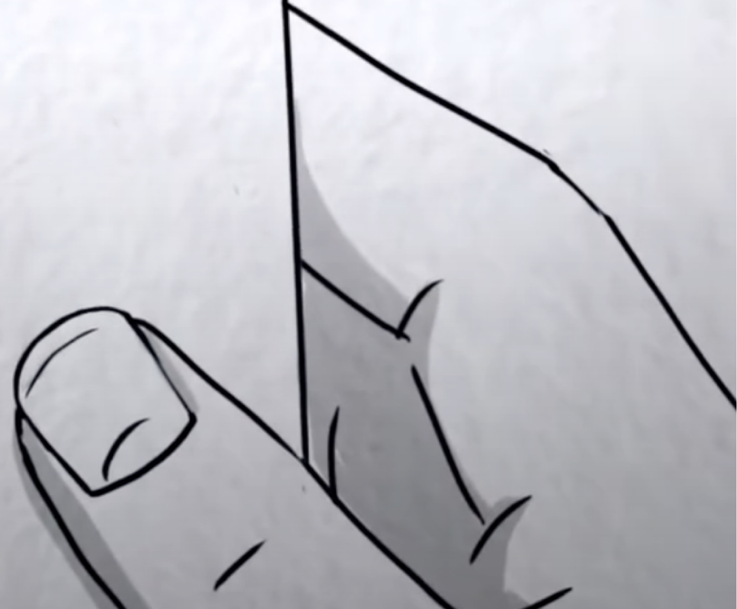

VIRAL GASTROENTERITIS

When should I bring my child to the Emergency Department?

1. Child is extremely tired
2. There are no tears when crying
3. No pee for about 12 hours
4. Overall more fluid coming out (vomiting/diarrhea) than going in (drinking)
5. Stomach pain not is not centred around the belly button

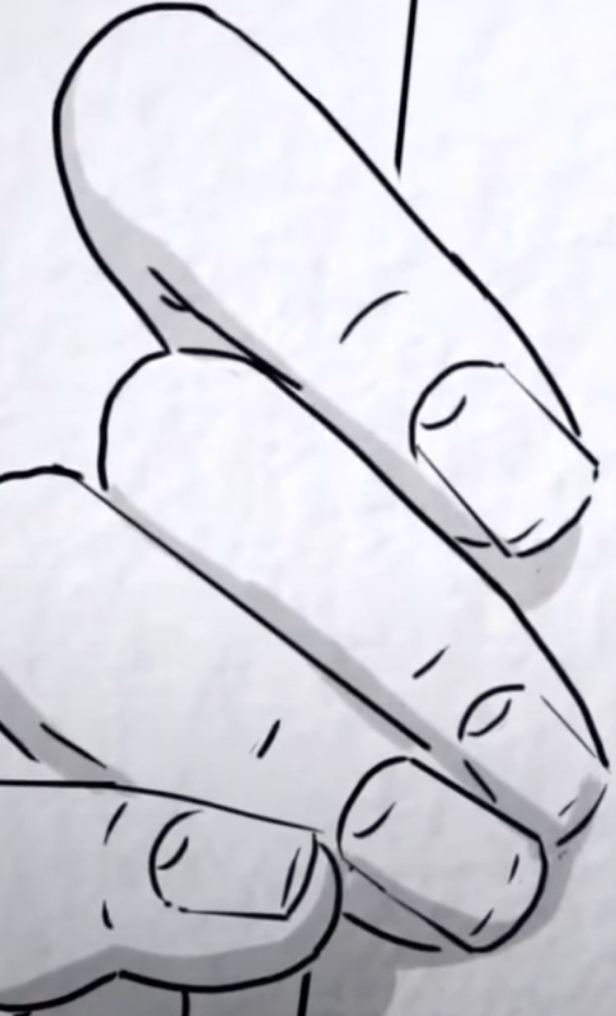

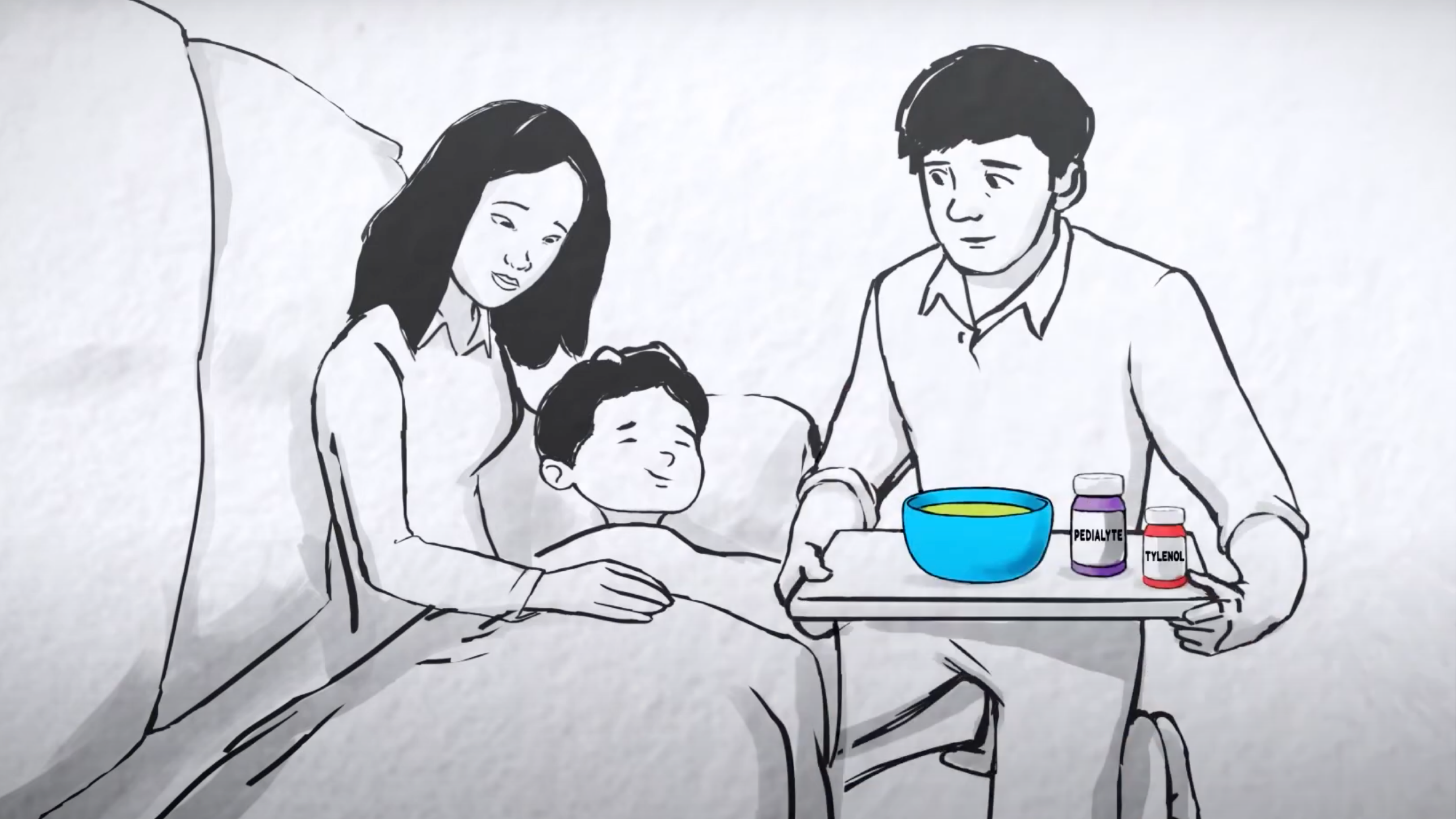
PEDIALYTE
TYLENOL

CHILDHOOD VOMITING AND DIARRHEA (GASTROENTERITIS)

A web-based knowledge translation tool for parents

Available at: https://www.echokt.ca/stomach-flu/

Developed under the leadership of Dr. Shannon Scott (Faculty of Nursing, University of Alberta) and Dr. Lisa Hartling (Department of Pediatrics, Faculty of Medicine & Dentistry, University of Alberta) with funding from the Canadian Institutes of Health Research

Dr. Scott is supported by a Canada Research Chair in Knowledge Translation in Child Health. Dr. Hartling is supported by a Canada Research Chair in Knowledge Synthesis and Translation. Dr. Scott and Dr. Hartling are Distinguished Researchers with the Stollery Science Lab supported by the Stollery Children's Hospital Foundation through the Women and Children's Health Research Institute

Funding

women & children's health research institute

STOLLERY CHILDREN'S HOSPITAL FOUNDATION

CIHR IRSC
Canadian Institutes of Health Research
Instituts de recherche en santé du Canada

Canada Research Chairs
www.chairs-chaires.gc.ca
