# Supplementary material for: Web-Based Knowledge Translation Tool About Pediatric Acute Gastroenteritis for Parents: Pilot Randomized Controlled Trial
Source: JMIR Form Res. 2023 May 25;7:e45276. doi: 10.2196/45276 (PMC10251226; doi:10.2196/45276)
Supplement: Multimedia Appendix 3 [file formative_v7i1e45276_app3.docx]

**Knowledge Question Scoring Framework:**

| Question | Answer (from video) | Correct numbers | Score |
| --- | --- | --- | --- |
| Fill in the blank: Gastroenteritis is often caused by __________ | a virus | text | virus=2,  Virus +bacteria/germ=1 anything else=0 |
| Dehydration is when: | more fluids are coming out than staying in | 2 | 2=1  anything else=0 |
| A child is likely dehydrated if he/she: | thirst, no tears when crying, sunken eyes, dry mouth, infrequent peeing, cold hands/feet, extreme tiredness | 1,3,4 | 1,3,4=2  partially correct=1  no correct answers=0 |
| You should take your child to the emergency department if he/she | 1. child is extremely tired, there are no tears when crying, no pee for 12 hours, overall more fluid coming out and in, stomach pain not centered around belly button, persistent dark green vomit, blood in diarrhea | 2,4,5 | 2,4,5=2  partially correct=1  no correct answers=0 |
| What types of fluids are encouraged to prevent/help dehydration? | clear fluids? | 4 | 4=1 |
| Which medications are helpful for a child with gastroenteritis? | tylenol | 1 | 1 only=2  partially correct, 1+something else,  no correct answer=0 |
| Fill in the blank: __________ is an example of a good oral rehydration solution to prevent and/or help dehydration. | pedialyte | text | 2=Pedialyte  1=Electrolyte  0-anything else |
| True of False. If your child is not dehydrated, but is vomiting and/or having diarrhea over a few days, you should take him/her to see a doctor | TRUE | 1 | 1=1  2=0 |

***Out of 13 total**
