# Supplementary material for: Web-Based Knowledge Translation Tool About Pediatric Acute Gastroenteritis for Parents: Pilot Randomized Controlled Trial
Source: JMIR Form Res. 2023 May 25;7:e45276. doi: 10.2196/45276 (PMC10251226; doi:10.2196/45276)
Supplement: Multimedia Appendix 4 [file formative_v7i1e45276_app4.docx]

**Decision Regret Scale***

Please think about your decision to bring your child to the hospital emergency department with vomiting and/or diarrhea.

Please show how you feel about these statements by moving the circle on the scale underneath your answer. Choose from 1 (strongly agree) to 5 (strongly disagree).


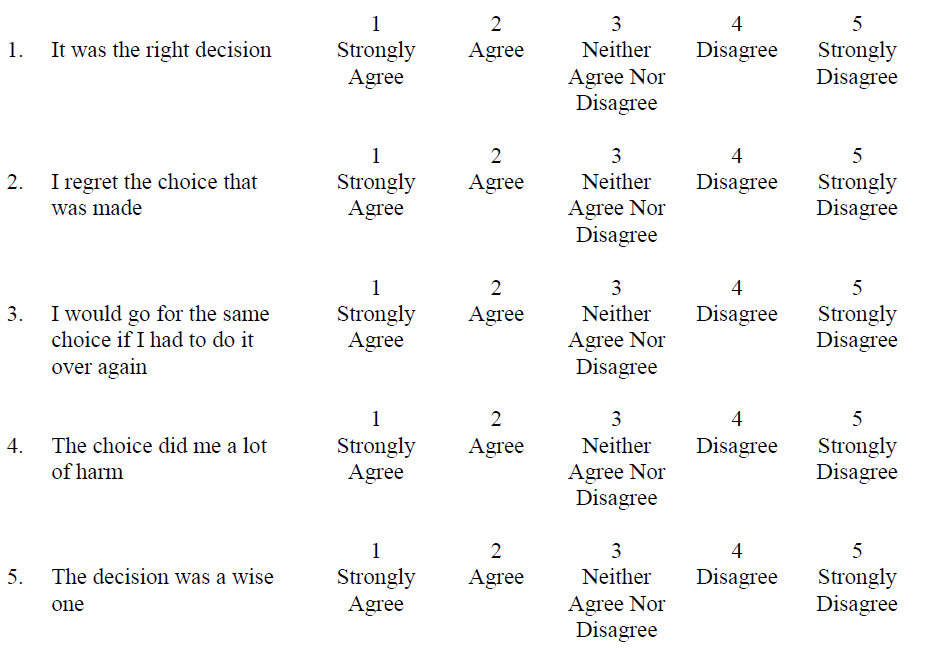


* From: The Ottawa Hospital. Decision Regret Scale. The Ottawa Hospital Research Institute. URL: https://decisionaid.ohri.ca/eval_regret.html
